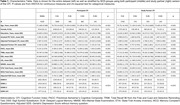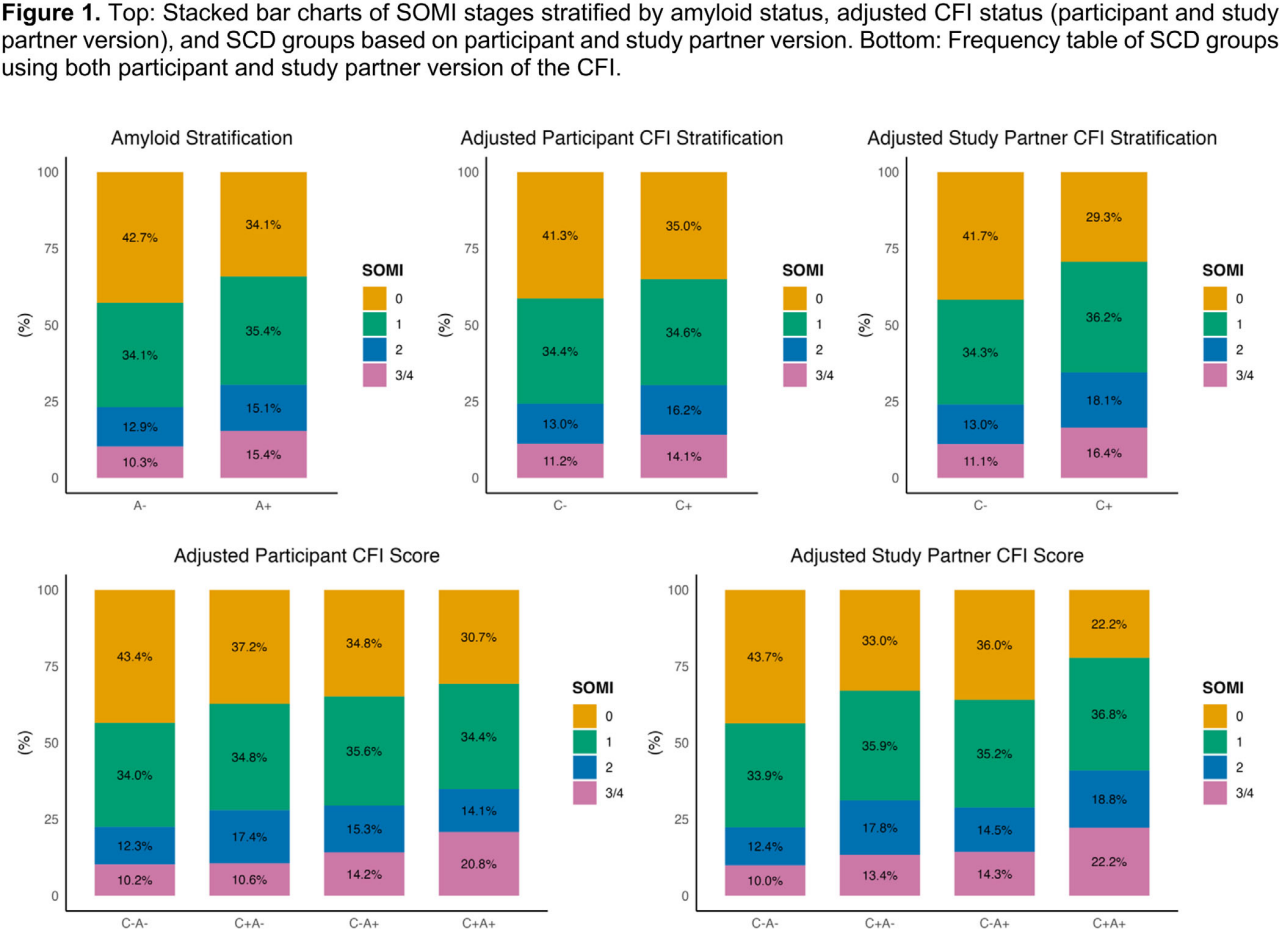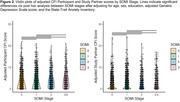# The relationship of subjective cognitive decline (SCD) and the Stages of Objective Memory Impairment (SOMI) system in preclinical AD

**DOI:** 10.1002/alz.093530

**Published:** 2025-01-03

**Authors:** Kellen K. Petersen, Laura Rabin, Ellen Grober

**Affiliations:** ^1^ Albert Einstein College of Medicine, Bronx, NY USA; ^2^ Brooklyn College of the City University of New York, Brooklyn, NY USA

## Abstract

**Background:**

Self‐awareness of declining cognition in the absence of objective cognitive impairment is called subjective cognitive decline (SCD). Herein, we examine the association of SCD measured by the Cognitive Function Index (CFI; Amariglio, et al. 2015) with the Stages of Objective Memory Impairment (SOMI) system (Grober, et al. 2018) that classifies cognitively normal individuals into one of five stages based on the Free and Cued Selective Reminding Test (FCSRT; Grober & Buschke, 1987) and that maps onto the presence of AD pathology (Grober, et al. 2022 & Petersen, et al. 2023). In early SOMI stages (SOMI‐1, 2) there is increasing retrieval difficulty; in later stages (SOMI‐3/4) storage deficits emerge.

**Methods:**

We used baseline data from 4109 neuropsychologically normal, older adults from the Anti‐Amyloid Anti‐Amyloid Treatment in Asymptomatic Alzheimer’s (A4) Study. All participants had amyloid PET measures and completed the FCSRT used for determing SOMI classification. Additionally, all participants and study partners completed the CFI. An adjusted CFI score was used that excluded three items that assess functional rather than cognitive ability. CFI positivity was defined by cutoffs, 7.8 and 6.5 for participant and partner versions respectfully, representing one standard deviation above the mean. Analysis of Variance (ANOVA) with post hoc analysis was used to assess differences of CFI scores between SOMI stages.

**Results:**

We found fewer A+ participants were SOMI‐0 compared to A‐ participants (34.1% vs. 42.7%, p<0.001, Figure 1). Additionally, more A+ participants were SOMI‐3/4 compared to A‐ participants (15.4% vs. 10.3%, p<0.001). Among A+ participants, memory impairment (SOMI‐2/3/4) was more prevalent among those with self‐perceived SCD (34.9%) than those without SCD (29.5%, p = 0.016). Similarly, among A+ participants, prevalence of memory impairment was higher among those with partner‐perceived SCD (40%) than those without (28%, p<0.001). Post hoc results found higher SOMI stages were associated with higher partner and study partner CFI scores except between SOMI‐2 and SOMI‐3/4 (p<0.05 for all, Figure 2).

**Conclusions:**

Memory impairment was found to be more prevalent among A+ participants with self‐ and partner‐rated SCD. SCD increases with increasing memory impairment.